# Taking care of the body to cure the mind: family-based therapies as attachment-based programs for adolescents with Anorexia Nervosa

**DOI:** 10.3389/fpsyg.2025.1710932

**Published:** 2026-01-13

**Authors:** Armando Cotugno, Francesca Manaresi, Valentina Cardi

**Affiliations:** 1 UOSD Eating Disorders Unit, ASL Roma 1, Rome, Italy; 2 Astrea Clinical Center, Circle of Security Parenting (COS-P) International, Rome, Italy; 3 Dipartimento di Psicologia Generale – DPG, Università degli Studi di Padova, Padua, Italy

**Keywords:** adolescents, Anorexia Nervosa, Attachment Theory, Circle of Security Parenting, eating disorders, Family-Based Treatment, parental empowerment

## Abstract

Anorexia Nervosa (AN) is a severe psychiatric disorder that typically emerges in adolescence and presents a high risk of medical complications and mortality. Early, family-centered interventions are critical in reducing both physical and psychological impairments associated with the illness. This article explores the clinical integration of Family-Based Treatment (FBT) with attachment-informed parenting interventions, particularly Circle of Security—Parenting (CoS-P). We argue that FBT can be conceptualized not only as a behavioral protocol for weight restoration but also as a structured framework for enhancing parental caregiving functions and emotional attunement. Drawing on developmental psychopathology and interpersonal motivational systems theory, we examine how FBT’s emphasis on externalization, parental empowerment, and family meals can be enriched through attachment-based strategies such as video feedback, emotional regulation, and reflective parenting. This integration offers a comprehensive treatment model aimed at restoring the adolescent’s developmental trajectory by supporting both embodied recovery and psychological growth within the family system.

## 1 Introduction

Anorexia Nervosa (AN) is a severe psychiatric disorder, characterized by a significant impairment in both physical health and psychosocial functioning (Treasure et al., 2020a). It is also one of the psychiatric conditions with the highest mortality rates. According to a review of 35 studies conducted by Arcelus et al. (2011), the standardized mortality rate (SMR) for AN is 5.86, indicating that individuals with this disorder are nearly six times more likely to die than the general population of the same age and gender. Importantly, treatment can significantly reduce this mortality risk. Without treatment, up to 20% of individuals with anorexia may die due to complications of the illness, whereas with adequate care, the mortality rate drops to 2–3%.

A key factor in treatment efficacy is early intervention, particularly when it occurs close to the onset of the disorder—which typically emerges in early adolescence. These considerations alone highlight the critical importance of disseminating evidence-based treatments within the psychiatric and medical communities. Psychotherapy remains the cornerstone of care for AN, even though international clinical guidelines emphasize that in cases involving malnourished patients, “the effectiveness of psychotherapeutic interventions remains uncertain” (American Psychiatric Association, 2005, 2023; NICE, 2004, 2017; Hay et al., 2014). This has led to the belief that psychotherapy can only be effective after malnutrition—the organic counterpart to the psychological dysfunction—has been addressed through residential rehabilitation or, in the most severe cases, hospitalization.

Family-Based Treatment (FBT) has emerged as an evidence-based outpatient strategy, applicable even to adolescents with moderate to severe malnutrition. FBT is recommended as the first-line outpatient treatment for patients who are medically stable and it has shown the ability to reduce the need for inpatient or residential care. The model is associated with fewer hospitalizations, faster achievement of full remission and weight restoration, and lower relapse rates (Lock et al., 2010). The opportunity to restore weight within the family context is fundamentally different from doing so in a hospital or residential setting.

FBT proceeds through three phases. In Phase 1, parents assume full responsibility for restoring regular eating and weight. Parents are supported to confidently protect the adolescent from the eating disorder while communicating emotional attunement rather than control, punishment, or anxiety. Because AN is egosyntonic and adolescents often resist nutritional recovery, prioritizing parental leadership over adolescent agency is crucial. Phase 2 introduces a developmentally attuned return of autonomy, supporting the child’s secure exploration. As the eating disorder relinquishes control, parents gradually hand back responsibility for eating, not as resignation but as a calibrated invitation: parents remain available as a secure base, monitoring risk, repairing ruptures, and adjusting boundaries when the adolescent shows signs of vulnerability or relapse. Phase 3 emphasizes healthy adolescent development beyond eating symptoms. The therapist guides the family to reflect on how the eating disorder previously disrupted psychological processes such as emotional regulation, dependence–independence balance, and parental protection. The therapist examines the adolescent’s developmental trajectory now that symptoms have subsided and supports the family in reinforcing relationships that enable secure autonomy.

In the attachment-integrated formulation we propose in the present article, FBT is conceptualized not only as a behavioral intervention aimed at nutritional rehabilitation, but as a structured clinical context in which parents are helped to resume their attachment functions as protective, emotionally attuned caregivers. Within this model, parental leadership during Phase 1 is framed as an expression of secure caregiving—where parents act as bigger, stronger, wise and kind figures who contain the eating disorder’s dysregulating influence while communicating connection and empathy rather than control, fear, or hostility. In this perspective, FBT facilitates a profound redefinition of the psychological and behavioral mechanisms underpinning family dynamics in high-demand relational contexts such as mealtimes, offering a therapeutic context that enables an emotionally corrective experience for all family members—producing effects that go well beyond the primary goal of the treatment (Cotugno, 2022; Lock and Le Grange, 2012).

## 2 Adolescence, embodiment, and Anorexia Nervosa

Any reflection on the psychotherapeutic treatment of AN must consider epidemiological data, which indicate that the highest incidence of this disorder occurs between the ages of 13 and 16 (Micali et al., 2013; Santomauro et al., 2021). In addition to the clinical features of AN, early intervention must also consider the dramatic changes that occur in this developmental phase, affecting both physical embodiment and neuropsychological structure.

*Adolescence is a pivotal stage in human development—second in importance* only to early childhood. It is marked by major psychological, physical, and relational transitions. The adolescent must progressively construct their adult identity through relationships with peers and novel life experiences, gradually separating from parental attachments while maintaining emotional connection. This developmental transition is supported by emerging psychological and relational skills, which are grounded neurobiologically in the process of frontal lobe maturation (Powell, 2006). Clinical and neuropsychological research has shown that malnutrition significantly disrupts this process: premorbid traits such as cognitive rigidity, hyper-attention to detail, difficulty with synthesis, and impaired processing of emotional states (e.g., fear, anger, sadness) tend to worsen in the context of malnutrition (Treasure et al., 2020b).

Although AN is often seen as a prime clinical lens for exploring the mind–body relationship, clinicians have traditionally interpreted the “anorexic body” symbolically, as a mentalized construct—rather than within a broader theoretical framework of the “embodied mind,” where perception, emotion, thought, and action are inseparable from the living, situated body (Borghi and Cimatti, 2010; Gallagher, 2007; Gallese and Sinigaglia, 2011; Merleau-Ponty, 1945).

Developmental psychopathology offers a compelling theoretical framework for understanding the role of the body across trajectories of continuity and change typical of adolescence (Cicchetti and Rogosch, 2002). The adolescent body becomes the catalyst for processes of identity construction that unfold in the delicate tension between “continuity and change”: “there is no other time in life when the body changes as dramatically as during puberty. These bodily changes are beyond the individual’s will or control, erupting and provoking intense emotional responses in the adolescent” (Diem-Wille and McQuade, 2021).

During this time of profound transformation, the body becomes an anchor—fragile and often illusory—onto which the adolescent clings to stabilize a shifting sense of self. Despite a pervasive sense of disorientation regarding values, desires, or life direction, the control of body shape and food intake may serve as a means through which individuals attempt to modulate and regulate their emotional experience. Evidence shows that during the COVID-19 pandemic there was a marked rise in body-related psychopathology among adolescents, particularly in non-suicidal self-injury and eating disorders. This increase underscores how the pandemic has tragically amplified the intricate interplay between developmental psychopathology, psychosocial context, emotion regulation processes and embodiment in adolescence (D’Agostino et al., 2022).

Considering findings from developmental psychopathology, FBT, which focuses on weight restoration and body care within the family system, should be viewed as a powerful therapeutic tool capable of reactivating the blocked developmental processes—perceptual, emotional, cognitive, and relational—disrupted by Anorexia Nervosa.

## 3 Malnutrition and family response

Critics of FBT often trivialize the emphasis it places on weight restoration—a central element of the approach—considering it an example of excessive medicalization that focuses too narrowly on behavioral and symptomatic aspects, rather than on the psychological mechanisms underlying eating disorders. However, the tight interconnection between malnutrition and psychological dysfunctions—such as food preoccupations, altered hunger and satiety regulation, heightened emotional distress, impaired cognitive functioning, and social withdrawal—was already evident in the Minnesota Starvation-Recovery Study conducted in the late 1940s (Keys et al., 1950).

A careful analysis of the results of the Minnesota Study laid the foundation for Garner’s pivotal contribution to the development of the cognitive-behavioral approach to AN (Garner et al., 1997; Garner and Bemis, 1982). The description of the “inevitable” perceptual, cognitive, emotional, and behavioral consequences of starvation led to a rethinking of certain features of the anorexic mental state—not as causes, but rather as effects of malnutrition. From this perspective, re-establishing biological and nutritional balance becomes essential for modifying cognitive-emotional distortions and restoring psychological functioning.

Malnutrition is not only the root of the adolescent’s physical and psychological deterioration in AN; it is also the central issue behind the family’s reaction to the illness. As early as the 19th century, Charles Lasègue had observed that the family’s response to anorexia constituted a major mechanism maintaining the anorexic behavior: *“The family has only two methods that it uses to the point of exhaustion: pleading and threatening… and the more their concern grows, the less appetite the patient has”* (Lasègue, 1873).

For decades, such observations led to viewing parents as responsible for their child’s anorexia, to the extent that separating the adolescent from the family (so-called “parentectomy”) was deemed a prerequisite for effective treatment (Charcot, 1885; Gull, 1874). In the 1960s and 1970s, pioneering work by Selvini-Palazzoli and later by Minuchin contributed significantly to developing family intervention protocols for treating AN (Minuchin et al., 1978; Selvini-Palazzoli, 1978). Minuchin famously described the characteristic communication patterns of “anorexic families” in terms of enmeshment and disengagement, concepts that closely resemble the *“pleading and threatening”* dynamic described by Lasègue. However, these family dynamics were still seen through a dominant paradigm that considered them etiological factors in the disorder.

It was only with research into Expressed Emotion (EE) (Leff and Vaughn, 1985) that a true paradigm shift occurred, allowing clinicians to understand family dynamics less as causes of the illness and more as consequences of AN’s impact on the family system.

The examination of families’ emotional responses to a child’s Anorexia Nervosa has revealed elevated levels of criticism and emotional overinvolvement (Kyriacou et al., 2007; Schmidt and Treasure, 2006). Such high Expressed Emotion can be understood as signaling a collapse of the parental caregiving system under conditions of threat. Elevated criticism and overinvolvement create relational climates marked by perceived danger, dysregulated caregiving, shame, and coercive parent–child cycles. These climates undermine parents’ ability to provide a safe-haven and secure-base stance—central in both FBT’s parental empowerment. As a result, the adolescent’s self-regulation capacities are compromised, physiological arousal increases, and core developmental and attachment needs are disrupted, thereby making weight restoration and psychological recovery substantially more difficult.

EE research once again confirmed Lasègue’s early insights: high parental emotional overinvolvement and criticism—the “pleading and threatening” described by Lasègue—appear to be the two key family response patterns linked to the maintenance of the disorder (Kyriacou et al., 2007; Schmidt and Treasure, 2006; Treasure et al., 2020b).

This paradigm shift in understanding the relationship between illness and parental behavior was consolidated in 2005 by Ivan Eisler, a family therapist at the Maudsley Hospital. In a seminal review on family interventions for AN, he noted: *“There is no doubt that the presence of an eating disorder has a significant impact on family life. Over time, food, eating, and associated concerns begin to saturate family interactions. Consequently, the family’s daily routines, as well as their coping and problem-solving behaviors, become disrupted”* (Eisler, 2005).

The typical dynamics observed in families with a child suffering from AN should therefore be seen primarily as reactions to the illness, rather than as causal mechanisms (Le Grange et al., 2010). The way family members respond plays a clinically significant role in the progression of serious psychiatric disorders (Leff and Vaughn, 1985).

FBT is a therapeutic approach explicitly inspired by EE research and is consistent with developmental psychopathology findings that highlight how adolescent separation–individuation processes are intrinsically tied to changes in parental caregiving. The adoption of a strong caregiving function—particularly, control over weight restoration—is central to FBT and aims to reduce the maintaining effects of the “inescapable” family response to AN. As previously noted, this response has been described across time in various ways by clinicians and researchers: *pleading vs. threatening* (Lasègue, 1873); *enmeshment vs. disengagement* (Minuchin et al., 1978); *emotional overinvolvement vs. criticism* (Leff and Vaughn, 1985). More recently, Treasure et al. (2016) have offered a more nuanced account of the communicative modalities through which family reactions are expressed. Their framework, emphasizing communication patterns, individual cognitive styles, and behavioral responses, appears conceptually aligned with the intervention model advanced in the present article.

We deliberately use the term *‘inescapable’* to characterize parents’ reactions to Anorexia Nervosa: in the next section, we argue that parental responses—especially emotional overinvolvement and criticism—seem, in many respects, to be intrinsically bound to the condition.

## 4 Family reaction and interpersonal motivational systems

Psychological and neurobiological research has shown that human interpersonal behavior is automatically regulated by innate Interpersonal Motivational Systems (IMS) (Liotti, 2001, 2005; Panksepp, 1998). These systems operate largely outside of conscious awareness and orient individuals toward relational goals with high adaptive significance. For the purposes of this discussion, we focus on three such systems: the Caregiving System (which supports the capacity to protect and nurture offspring), the Defense System (which organizes protective responses to perceived threats), and the Attachment System (which governs help-seeking behaviors in states of vulnerability).

Even from simple ethological observation, we know that when a threat to offspring is perceived, parental caregiving behavior is guided by two primary motivations: protecting the child from danger (Caregiving System) and attacking the threat (Primary Defense System). In such scenarios, these two motivational systems produce coherent and integrated behavior—caring for the child while neutralizing the external danger.

However, in Anorexia Nervosa, the parent or caregiver is caught in an inescapable relational paradox: the child is perceived as being in danger (malnourished and weak), but the source of that danger is the very behavior (food restriction) enacted by the child. In other words, the adolescent is automatically perceived as both victim and threat. This paradox leads to the simultaneous activation of both caregiving and defensive motivational systems, but without the possibility of integrating them into a coherent response. Instead, they clash, producing a disorganized oscillation between aggressive and caregiving behaviors.

This motivational conflict helps explain why 19th-century psychiatrists deemed families “unfit” to care for individuals with anorexia, and why parentectomy was proposed as a therapeutic strategy—to reduce the harm caused by what we now recognize as a cognitive-interpersonal maintenance cycle (Safran and Segal, 1990). Yet even Lasègue (1873), with remarkable clinical insight, emphasized the importance of understanding *“the illness of the anorexic patient always in parallel with the anxieties and concerns of her family,”* highlighting the need to help parents move beyond the use of *“pleading and threatening.”*

More than a century after Lasègue’s observations, the work conducted by the Eating Disorders Unit at the Maudsley Hospital and the clinical research by Lock and Le Grange (2012) led to the development of a series of therapeutic techniques designed to reactivate the parents’ caregiving abilities to support the adolescent’s healthy autonomy and guide them back onto their developmental path.

This approach—especially in how it assigns parents an active and competent role in the process of refeeding—stands in sharp contrast to older treatment models for adolescent AN, which framed the disorder as a problem of individuation (Smolak and Levine, 1993). Those theories viewed family dynamics as etiologically implicated in anorexia and therefore promoted physical and emotional separation from a presumed “anorexogenic” environment.

In contrast, FBT’s emphasis on weight restoration within the family brings about a different kind of recovery—one that fosters not just physical improvement, but also individual and relational psychological change. These changes are achieved not through prescriptive techniques, but through a collaborative search for new problem-solving strategies.

FBT aims to interrupt the interpersonal maintenance cycle of AN by helping parents develop improved metacognitive and emotional regulation skills—thereby reducing the automatic behavioral reactions rooted in biologically driven motivational systems. One of FBT’s most effective tools in this regard is psychoeducation: helping parents *see the illness* and distinguish it from their child’s developmental needs.

This conceptual framework underlies one of the key therapeutic strategies in the FBT model: externalization of the anorexic illness. Externalization promotes parental mentalization—facilitating understanding of the dysfunctional psychological mechanisms underlying AN. As reflective capacity improves, so does emotional regulation, enabling parents to provide developmentally appropriate caregiving support.

By distinguishing “anorexic thoughts and behaviors” from the adolescent’s basic developmental needs, externalization allows parents to temporarily take responsibility for fulfilling those needs when the adolescent is unable to do so due to the disorder. The *externalization* of the Anorexia Nervosa is one of the five core principles on which the FBT protocol is based, briefly summarized below:

1. *Agnostic view of the origin of the disorder.* The therapist does not take a position on the cause of the eating disorder, avoiding attributing blame to either the parents or the adolescent. This approach reduces family conflict and facilitates the therapeutic alliance.
2. *Collaborative role of the therapist*. The therapist adopts a collaborative stance and guides the process without imposing solutions. This fosters active involvement from both parents and the adolescent in building the treatment path.
3. *Parental empowerment*. Parents are seen as the most important resource for their child’s recovery. They are entrusted with the responsibility of leading refeeding and supporting the adolescent’s psychophysical restoration.
4. *Externalization of the disorder*. As mentioned, this allows the family to “fight” the anorexia rather than the adolescent, thereby reducing guilt and resistance to treatment.
5. *Pragmatic approach.* FBT favors practical, goal-oriented interventions, initially focusing on weight restoration and addressing deeper psychological aspects only at a later stage.

These five principles form the backbone of the FBT protocol and account for its strength and effectiveness in the treatment of adolescent eating disorders (Ellison et al., 2012; Rienecke and Le Grange, 2022). As we previously described, FBT is structured in three progressive phases: in the *Phase 1*, the therapist helps parents take responsibility for restoring the adolescent’s weight; in the *Phase 2*, control over eating is gradually returned to the adolescent; and in the *Phase 3*, the developmental tasks characteristic of adolescence are briefly discussed and, where necessary, this may serve as a prelude to individual treatment. Each phase has specific therapeutic goals and interventions. In the next paragraph, we will explore possible integrations of the FBT protocol with parenting empowerment interventions inspired by clinical research on attachment.

## 5 FBT and parental empowerment: an integration with Attachment Theory

The FBT protocol shares numerous theoretical and pragmatic affinities with parenting empowerment interventions grounded in Attachment Theory, such as Circle of Security Parenting (COS-P) (Powell et al., 2016; Pazzagli et al., 2014) and the Connect – Attachment-Based Program for Adolescents (Moretti et al., 1994; Moore et al., 1998; Moretti et al., 2002). Drawing on research showing that parental support is a significant protective factor in adolescent development, these programs aim to reduce dysfunctional adolescent behaviors by strengthening caregivers’ capacity to provide effective care. These approaches are oriented toward ensuring the conditions of personal safety necessary to sustain the differentiation and individuation processes that define the adolescent transition. Both clinical applications of Attachment Theory and findings from Developmental Psychopathology emphasize the need to balance therapeutic interventions that promote separation and individuation with those that ensure emotional safety, which is essential for the adolescent’s healthy progression toward autonomy.

This clinical-theoretical perspective aligns closely with FBT, whose hierarchical structure begins by actively supporting parental caregiving capacities (Phase 1) and gradually promotes the adolescent’s autonomy in managing their eating behaviors (Phases 2 and 3).

The central hypothesis of this article is that the core principles of FBT can be easily integrated with key concepts from Attachment-based clinical research. This is not merely a speculative claim; rather, the goal is to incorporate attachment-informed clinical practices into the FBT protocol—particularly in Phase 1, which establishes the foundation for collaborative parental involvement in helping the adolescent restore adequate eating behaviors and physiological weight. From the very first sessions, the therapist helps parents recognize that their child’s restrictive eating is not simply a matter of stubbornness, vanity, or a desire for control. Rather, it is a deeply maladaptive coping strategy that the adolescent uses to manage intense emotional discomfort (Fitzsimmons and Bardone-Cone, 2010). Restricting food and focusing obsessively on weight and shape give the adolescent a temporary—though ultimately harmful—sense of stability and mastery. In other words, the act of controlling the body through dietary restriction becomes a way to regulate overwhelming feelings and to reduce inner vulnerability when they lack more adaptive strategies for self-soothing and emotion regulation (Dougherty et al., 2023; D’Agostino et al., 2022).

By making this psychological mechanism explicit, the therapist helps parents differentiate between the adolescent’s developmental needs (such as the search for safety and self-coherence) and the symptomatic anorexic behavior (including restrictive thoughts and actions). This externalization becomes the basis for improving parental reflective functioning—or mind-mindedness—and for engaging them in collaborative support aimed at helping the adolescent overcome emotional difficulties through a pragmatic, problem-solving approach.

The founding principles of FBT, as previously outlined, can be naturally integrated with the operational guidelines of Circle of Security – Parenting (COS-P) (Cotugno, 2018). COS-P is a program designed to help parents better understand their children’s emotional needs and respond to them more effectively: its aim is to provide the parents with the following skills:

- Accurate observation of the adolescent and family interactions.
- Distinction between observed data and interpretations of relational phenomena.
- Reflection on parental psychological dimensions that influence caregiving behavior.
- Identification and support of effective caregiving behaviors, especially those addressing the child’s emotional needs.
- Activation of reparative strategies in caregiving.

COS-P is based on four fundamental principles:

1. *Protection*: Parents are guided to provide a secure base from which the adolescent can resume the exploration process that has been stalled by anorexia. This involves balancing the adolescent’s need for autonomy and differentiation with the need to provide a safe and protected environment.
2. *Relief*: Parents are supported in becoming a secure base the adolescent can rely on during moments of distress. Emotional availability means being able to listen empathetically and respond to the child’s feelings and concerns.
3. *Emotion regulation*: Parents are taught how to understand and regulate their child’s emotional distress. This includes modeling effective coping strategies and offering emotional guidance.
4. *Circle of security map*: This graphic representation illustrates the structural dynamics that govern the interaction of four motivational systems—Attachment, Caregiving, Defense, and Exploration (Liotti, 2001; Manaresi, 2015).

The natural flow of the adolescent’s lived experience—which supports the integration of autonomy, exploration, and emotional security—is disrupted by Anorexia Nervosa. Involving families according to COS-P principles enhances FBT interventions aimed at empowering parents to take charge of weight restoration and support adequate eating behavior in their child.

The “hands” metaphor is central to both the FBT and COS-P models. In FBT, encouraging parents to “take matters into their own hands” when it comes to restoring their adolescent’s weight means guiding them to assume a concrete role in emotional containment and organization—particularly during meals, which are often the most emotionally intense moments in therapy. This process often triggers high levels of anxiety in parents, which must be mobilized to facilitate interventions counteracting the adolescent’s symptomatic behaviors. Although effective, FBT is often experienced as demanding for both parents and adolescents (Parent and Parent, 2008). Parents are expected to make significant sacrifices—such as preparing and supervising all meals—and to maintain strict boundaries to counteract the eating disorder’s manipulations. At the same time, adolescents may perceive this parental control as oppressive or inappropriate for their age (Murray et al., 2012). Through psychoeducational sessions, parents learn the importance of responding empathetically and non-judgmentally to the challenges their adolescent is facing. This includes acquiring knowledge about the causes and dynamics of anorexia—biological, psychological, and social—and developing the emotional presence and reassurance needed during moments of crisis. By integrating the COS-P principles, FBT’s “hands” metaphor becomes more flexible and targeted in addressing emotional difficulties. Therapists support parents not only in managing anorexia-related behaviors, but also in regulating their own emotional reactions to their child’s dysfunction. This dual focus results in a comprehensive educational process—not just about the eating disorder, but about how parents interact with their children daily. The therapist helps them strike a delicate balance between firm support for adaptive eating behaviors and empathic attunement to the adolescent’s emotional challenges.

In turn, the therapist—through an empathic and collaborative stance—acts as a “pair of hands” for the parents, holding and containing their emotional struggles while supporting their caregiving efforts for the adolescent.

## 6 Video-feedback and the operational integration of FBT and CoS-P

A clear example of the operational value of integrating FBT with COS-P can be found in the use of the Circle of Security map to manage the psychological and interpersonal dynamics that arise during mealtimes. The family meal session is the core therapeutic intervention in Phase 1 of FBT. It provides the therapist with a powerful tool for observing and modifying the family’s response patterns, which often act as key interpersonal maintenance mechanism Anorexia Nervosa. During the family meal session, the therapist actively supports the parents as they help their child “eat a little more.” The disruptive role of the therapist’s intervention is precisely what allows for direct work on the dynamics of overinvolvement, criticism, and helpless anger, which are typically activated by the challenge of getting the adolescent to eat.

Video-feedback, a technique widely used in COS-P, can dramatically amplify the therapeutic effectiveness of the family meal session. In our clinical experience, joint viewing of the video recording—between therapist, parents, and adolescent—facilitates reflective observation and strengthens the therapeutic meaning of what occurred during the session. Through joint observation, the therapist helps the family identify the chain of behaviors, emotions, and thoughts that unintentionally reinforce oppositional, fearful, or restrictive behaviors in the adolescent. The video-feedback process becomes a powerful tool for analyzing and modifying cognitive-interpersonal maintenance mechanisms—which are typically driven by the automatic activation of specific Interpersonal Motivational Systems (Attachment, Caregiving, Defense, and Exploration). The “slow-motion replay,” enabled by video-feedback, makes it possible to mentalize the underlying emotional, cognitive, and behavioral processes, allowing both parents and adolescent to step out of dysfunctional automatic responses and begin to build more effective regulation strategies.

The video recording of the family meal session is first reviewed by the therapist using the COS-P categories previously described. It is then examined together with the parents and the adolescent through a structured sequence of three joint review phases. The first phase consists of the therapist and the adolescent watching selected segments together; this is followed by a second phase in which the therapist reviews the video with the parents. The final step involves a joint discussion of the video sequences with both the parents and the adolescent present. The video-feedback session typically lasts between 90 and 120 min.

The integration of FBT and COS-P finds in video-feedback a highly effective operational synthesis, enabling parents to:

- better understand the psychological characteristics of anorexia and distinguish them from their child’s developmental needs;
- improve caregiving abilities in support of their adolescent’s emotional self-regulation;
- restore a sense of parental efficacy.

From the adolescent’s perspective, this integration supports the ability to:

- distance and differentiate themselves from anorexic mental processes;
- recognize how the “anorexic thinking” contributes to dysfunctional family dynamics;
- access more adaptive and effective coping strategies.

By activating “hot cognitions” during the family meal session and carefully analyzing the interactional dynamics through video feedback, the COS-P/FBT integration allows for:

- a better understanding of motivational transitions between Attachment, Caregiving, Defense and Exploration systems;
- emotion regulation through empathic support of the adolescent’s internal;
- in-depth exploration of specific roles and mental states within the family’s interactive patterns.

The reflective exploration of parents’ and adolescent’s mental states during the video-feedback session creates a shared reference scene, which can be mapped using the COS-P framework. This mapping helps build more effective coping strategies for both adolescent and caregivers.

The implementation of alternative and more functional strategies is central throughout Phase 1 of the FBT protocol. The integration of COS-P principles enables the therapist to intervene with greater flexibility, adapting therapeutic work to the specific psychological characteristics of each family. The ongoing exploration of intrapersonal and interpersonal processes, guided by the COS-P framework, lays the groundwork for Phase 2 of FBT, in which control over food is gradually returned to the adolescent. Supporting the adolescent in exploring new coping strategies—both within the family and in broader social contexts—is the focus of Phase 2 and leads into Phase 3, where developmental tasks typical of adolescence are addressed. When necessary, this final phase may include individual psychotherapeutic work, aimed at exploring the adolescent’s psychological difficulties that have become more evident during FBT (e.g., dysfunctional beliefs, emotional functioning, interpersonal issues, and emotion regulation).

## 7 Conclusion

The treatment of Anorexia Nervosa (AN) requires a multidisciplinary approach involving mental health professionals, nutritionists, and medical doctors. Across different clinical models, there is broad consensus on the central role of family involvement in the treatment of adolescent eating disorders (Deci and Ryan, 2008; Klein and Miller, 2021; Miller et al., 2007; Moore et al., 1998; Moretti et al., 2002; Pazzagli et al., 2014; Treasure et al., 2016). Family-Based Treatment (FBT) represents the most extensively validated early intervention protocol for adolescent AN, with multiple studies confirming its efficacy in improving eating disorder symptoms, enhancing quality of life, and strengthening caregivers’ sense of competence (Lock et al., 2010; Le Grange et al., 2012). Nevertheless, while evidence for FBT is robust, full remission remains difficult to achieve for more than half of patients. Preliminary studies on treatment moderators offer promising directions, suggesting the possibility of identifying patient subgroups most, or least, responsive to this model (Lock and Le Grange, 2019). Improving outcomes in adolescent AN remains a clinical and public health priority, as more effective interventions can save lives, prevent chronicity, and sustain long-term developmental recovery.

### 7.1 Beyond standard FBT: adaptations and innovations

In light of these challenges, several adaptations of FBT have been developed to respond to different clinical profiles, including adolescents with bulimia nervosa, high emotional dysregulation, and impulse control difficulties (Loeb et al., 2020; Murray et al., 2015; Peterson et al., 2019; Rathus and Miller, 2014; Torneke et al., 2022). One recent line of work has shown that intensive parental coaching may improve outcomes for adolescents with AN whose parents report low self-efficacy (Lock et al., 2024). Parental coaching resonates with the theoretical underpinnings of the New Maudsley Method, which complements the behavioral emphasis of FBT by integrating attention to parents’ cognitive-emotional processes and communication styles (Treasure et al., 2016). This approach highlights how specific patterns of communication and parental responses may, often unintentionally, accommodate or reinforce the illness. The aim of the New Maudsley Method framework is therefore twofold: to reduce family members’ anxiety and distress, and to provide carers with skills, techniques, and communication tools that facilitate engagement with the adolescent, enhance their self-esteem, and cultivate resilience as a foundation for change.

### 7.2 Integrating FBT and attachment-based parenting interventions

In continuity with these developments, the present article advances a clinical-theoretical integration between FBT and parenting empowerment interventions grounded in Attachment Theory. More specifically, it illustrates the operational connections between FBT and Circle of Security Parenting (CoS-P), offering a framework that underscores the deep continuity between bodily and psychological care in adolescence.

This perspective highlights the importance of interventions that foster parental reflective functioning and empathic attunement to emotional distress. The emphasis on parental mentalization (mind-mindedness) translates into concrete therapeutic practices that help parents distinguish the adolescent’s identity from the restrictive, cognitive-behavioral patterns imposed by the eating disorder. In this sense, parental responsibility for weight restoration becomes inseparable from the creation of secure attachment conditions, where the restoration of the body is conceived as a pathway to psychological healing.

Clinical tools such as video feedback—widely used in parenting empowerment programs—illustrate how therapists can simultaneously support parents in managing their own emotional responses and help them remain actively engaged in addressing the adolescent’s distress. Such interventions expand the therapeutic focus beyond weight restoration alone, foregrounding the adolescent’s psychological suffering and the developmental challenges embedded in their recovery process. Giving priority to the adolescent’s voice and identity negotiations is crucial, as these elements mediate how families and clinicians together address the physical crisis of AN while fostering a more holistic and durable recovery.

Ultimately, the hierarchical structure of treatment situates physical recovery as the central and non-negotiable first step. Yet, through a delicate balance of emotional validation, reflective dialogue, and the reinforcement of adaptive coping strategies, treatment can also re-establish the adolescent’s natural developmental trajectory. In this integrated perspective, bodily care and psychological care are no longer separate domains but rather complementary pathways, converging toward the adolescent’s recovery, resilience, and capacity for growth.

## Data Availability

The original contributions presented in the study are included in the article/supplementary material, further inquiries can be directed to the corresponding author.
